# Machine Learning in Acute Ischemic Stroke Neuroimaging

**DOI:** 10.3389/fneur.2018.00945

**Published:** 2018-11-08

**Authors:** Haris Kamal, Victor Lopez, Sunil A. Sheth

**Affiliations:** Department of Neurology, University of Texas at Houston Health Science Center, Houston, TX, United States

**Keywords:** stroke, neuroimaging, machine learning (artificial intelligence), neurosciences, support vector machina (SVM), stroke management, stroke diagnosis

## Abstract

Machine Learning (ML) through pattern recognition algorithms is currently becoming an essential aid for the diagnosis, treatment, and prediction of complications and patient outcomes in a number of neurological diseases. The evaluation and treatment of Acute Ischemic Stroke (AIS) have experienced a significant advancement over the past few years, increasingly requiring the use of neuroimaging for decision-making. In this review, we offer an insight into the recent developments and applications of ML in neuroimaging focusing on acute ischemic stroke.

Machine Learning (ML), considered a branch of artificial intelligence, is a field of computer science and engineering that facilitates extraction of data based on pattern recognition. A computer learns from previous mistakes after repeated analysis of data and masters tasks that were previously considered too complex for a machine to process (1). The development of these systems to interpret data in neuroimaging has provided valuable information for research in matters of the interaction, structure, and mechanisms of the brain and behavior in certain neurological disorders (2, 3).

Machine learning systems are now being implemented in the clinical neurosciences to devise imaging-based diagnostic and classification systems of neoplasms of the brain (4–6), certain psychiatric disorders (7–11), epilepsy (12, 13), neurodegenerative disorders (14–20), and demyelinating disorders (21–23). In this review, we discuss the present-day role of ML focusing on acute ischemic stroke (AIS), discussing its potential and limitations.

## Machine learning in the clinical neurosciences

The use of neuroimaging in the evaluation of many neurological diseases such as dementia, epilepsy, demyelinating diseases, depression, and schizophrenia has grown tremendously. This burgeoning interest has been met with an expansion of ML algorithms in neurosciences (1, 24).

Oliveira et al. (14) evaluated an unsupervised ν-One-Class Support Vector Machine (ν-OC-SVM) trained with neuroimaging variables, such as cortical thickness and cerebral volume of the brain, from healthy subjects to calculate an abnormality index and compare it with patients diagnosed with mild cognitive impairment (MCI) and Alzheimer's disease (AD). The method correctly classified AD subjects as outliers with an accuracy of 84.3%, and the brain abnormality index was directly associated with the group diagnosis, clinical data, biomarkers, and risk of future conversion to AD.

In schizophrenia, Greenstein et al. (9) used Random Forest (RF), a machine learning algorithm, to discriminate between childhood-onset schizophrenia and healthy patients based on brain magnetic resonance imaging (MRI) measurements of regions of interest (ROI): left temporal lobes, bilateral dorsolateral prefrontal regions, and left medial parietal lobes. The algorithm correctly classified groups with 73.7% accuracy, and a greater brain-based probability of illness was associated with a statistically significant worse functioning and fewer developmental delays. Machine learning can also help distinguish between subsets of a certain disease. Bleich-Cohen et al. (7) utilized Searchlight Based Feature Extraction (SBFE), a data-driven multi-voxel pattern analysis (MVPA) approach, to search for activation clusters of cognitive loads in brain functional Magnetic Resonance Imaging (fMRI). This ML method helped to identify the two subgroups of schizophrenic patients with and without Obsessive-Compulsive Disorder (OCD) with a 91% accuracy, successfully delineating between symptom severity and a psychiatric comorbidity.

An et al. (12) compared whole-brain white matter changes in patients with mesial temporal epilepsy and matched healthy controls, evaluating tract-based spatial statistics and fractional anisotropy with an ML approach. This ML-based approach discriminated each group accurately and demonstrated high sensitivity to changes in fractional anisotropy in mesial temporal epilepsy patients, which may be beneficial when no lesion can be identified on neuroimaging. Moghim et al. (13) introduced a predictive model for seizure occurrence in a single patient. This approach was based on a multi-class support vector machine (SVM) and 14 selected features of an electroencephalogram in patients with epilepsy. The predicted time of seizure with a window between 20 and 25 min was reported with an average sensitivity of 90.15, 99.44% specificity, and 97% accuracy.

Lesion burden estimation in traumatic brain injury (TBI), AIS, dementia, and multiple sclerosis serves to identify the affected regions, the extent of damage, and therefore, the functional outcome in such patients. Kaminatas et al. (25) proposed an approach for lesion segmentation using a multi-modal brain MRI based on an 11-layers deep, multi-scale, 3D Convolutional Neural Networks (CNN) called Deep Medic. Their proposed novel training scheme is based on two main components, a 3D CNN that produces accurate soft segmentation maps and a connected Conditional Random Field that imposes regularization constraints on the CNN output and produces the final hard segmentation labels. This allows for a deeper and more discriminative delimitation of lesion burden, with the highest reported accuracy observed in a cohort of patients with severe TBI.

## Challenges in acute ischemic stroke

Stroke is the leading cause of serious long-term disability and the fifth leading cause of death in the United States, with its prevalence increasing with advancing age in both males and females, as each year ~795,000 Americans experience a new or recurrent stroke (26). This burden is coupled with a direct medical expense of an estimated $23.6 billion according to the last annual report of 2014 (26). With the increasing complexity of the acute ischemic stroke therapy and the rising of per-person costs, there is a real and urgent need for a technological solution to aid in the streamlined care of patients and selection of the appropriate therapeutic intervention.

Present treatments for AIS revolve around rapid reperfusion of ischemic tissue, using intravenous (IV) thrombolytic medications such as tissue plasminogen activator (tPA) and/or endovascular techniques to mechanically remove the obstruction to blood flow. Contemporary clinical trials are now implementing a higher complexity of neuroimaging modalities to define treatment standards, resulting in an increased economic as well as logistical burden on healthcare. The WAKE-UP multicenter clinical trial (27) used magnetic resonance imaging (MRI) in patients that presented with an unknown time of onset of symptoms to identify brain regions that exhibited a restricted diffusion on diffusion-weighted imaging (DWI) scan and no T2-signal hyperintensity on fluid-attenuated inversion recovery (FLAIR) sequence, estimating the onset of the infarct to be < 4.5 h and thus guiding stroke therapy. Previous to this study, non-contrast head CT, an imaging modality that is widely and readily available, was the only imaging screen used to assess for tPA eligibility. The new, expanded tPA indication requiring MRI poses challenges for a majority of centers, which do not have ready access to this type of imaging emergently and 24/7.

The growing dependence on neuroimaging in determining treatment options for acute ischemic stroke is observed as well for endovascular stroke therapy (EST), which has shown to improve outcome when used in combination with standard medical care (28). In 2015, numerous clinical trials demonstrated a clear benefit of endovascular treatment over medical management alone for a select group of patients with acute ischemic stroke seen within 6 h of the onset of stroke (29–33), and relied on imaging modalities including NCHCT, CT/MR angiography (CTA/MRA) and CT/MRI perfusion (CTP/MRP) scans. Results derived from these trials showed an advantage in using advanced imaging modalities in identifying patients with a higher likelihood of better outcomes from EST. Two additional clinical trials (34), DAWN and DEFUSE3, published in 2018 evaluated a much larger population of stroke patients, those presenting up to 24 h after their symptoms, and required the use of perfusion imaging with CT or MRI.

This increased reliance on neuroimaging has led to a tremendous improvement in our ability to care for patients with AIS but has been coupled with a number of challenges. Specifically, limited availability of these imaging modalities, a shortage of specialists to promptly interpret these studies, as well as inter-observer variability have limited the implementation of the above findings. Indeed, studies evaluating inter-observer performance on Alberta Stroke Program Early CT Score (ASPECTS), a 10-region imaging grading system in stroke, showed significant variability (35–39). Further adding to the complexity of acute stroke treatment is that while the need to perform and interpret advanced neuroimaging has recently increased, the urgency with which such evaluation is being performed has remained the same. For every minute that a patient with a large vessel occlusion fails to be treated, an estimated 1.9 million neurons and 14 billion synapses are lost in the brain (40). Trials evaluating efforts to promptly assess and treat patients with AIS have demonstrated superior outcomes and decreased morbidity. In patients treated with intravenous thrombolysis, reducing treatment times by 15-min was associated with reduced in-hospital mortality, reduced incidence of symptomatic intracranial hemorrhage, and a greater likelihood of independent ambulation at discharge (41, 42). In patients treated with endovascular therapy, for every 15-min reduction of onset to recanalization of the occluded artery, 34 per 1,000 treated patients had improved disability outcome (43). As such, there is an urgent need for systems to rapidly and precisely interpret neuroimaging data in acute ischemic stroke.

## Implementations of machine learning in acute ischemic stroke

Machine learning algorithms have been used to assist in the diagnosis and individualized treatment decisions in acute ischemic stroke. The implementations of machine learning are numerous, from early identification of imaging diagnostic findings (44), estimating time of onset (27, 45), lesion segmentation (46), and fate of salvageable tissue (47, 48), to the analysis of cerebral edema (49, 50), and predicting complications (51–53) and patient outcomes (54–57) after treatment. A summary of the most recent articles investigating the applications of machine learning for automated diagnosis and outcome prediction in acute ischemic stroke is given in Table 1.

**Table 1:** Use of machine learning in stroke diagnosis and outcome prognosis.

**References**	**ML-based approach**	**Feature**	Results^*^
Asadi et al. (55)	Artificial neural network	Prediction of Dichotomized mRS	70% accuracy
Bentley et al. (51)	Supported vector machine	Prediction of sICH	74.4% accuracy
Bouts et al. (47)	Adaptive boosting	Prediction of infarction volume	89 ± 5% accuracy
Chen et al. (46)	RF + GAC	Relation of CSF shifts and cerebral edema	*r* = 0.879
Forkert et al. (56)	Multi-class supported vector machine	Predicted 30-day post-stroke mRS
		*Multi-value mRS*	56% accuracy
		*Multi-value mRS±1*	82% accuracy
		*Dichotomized mRS*	85% accuracy
Huang et al. (48)	Supported vector machine	Predicted infarct penumbra volume
		*30-min occlusion of MCA*	86 ± 2.7% accuracy
		*60-min occlusion of MCA*	89 ± 1.4% accuracy
		*Permanent occlusion of MCA*	93% accuracy
Scalzo et al. (52)	Non-linear regression model	Prediction of HT	>85% accuracy
Takahashi et al. (44)	Supported vector machine	Detection of MCA dot sign	97.5% sensitivity
Yu et al. (53)	SR-KDA	Prediction of HT	83.7 ± 2.6% accuracy
Nielsen et al. (54)	Deep features CNN	Prediction of patient outcome after IV thrombolysis	88 ± 0.12% accuracy

* *The results displayed for each article are the most accurate or relevant in matter of the machine learning approach utilized according to the author*.

*CNN, Convoluted Neural Network; GAC, Geodesic Active Contour; HT, Hemorrhagic transformation; MCA, Middle Cerebral Artery; mRS, modified Rankin Scale; RF, Rain Forest; sICH, symptomatic Intracranial Hemorrhage; SR-KDA, Spectral Regression Kernel Discriminant Analysis*.

One of the most relevant clinical criteria to decide if a patient with an acute ischemic stroke is eligible for IV thrombolysis with tPA is a time from symptom onset of < 4.5 h, but in medical practice, stroke symptom onset is usually unknown. Ho et al. (45) developed a deep learning algorithm based on an autoencoder architecture to extract imaging features in perfusion-weighted images (PWI) in MRI to determine the time elapsed since stroke onset.

Lesion estimation and identification of salvageable tissue are essential in the acute decision making in stroke, but the expense and resources involved present a challenge for physicians. Chen et al. (46) used a framework with two CNNs to segment stroke lesions using DWI in MRI. One CNN was a combination of two DeconvNets (EDD Net), and the second CNN was a multi-scale convolutional label evaluation net (MUSCLE Net) to help reduce the potential false positives detected by the EDD Net. The dataset was built with clinical acquired DWI from 741 subjects, exhibiting a high lesion detection rate, and accuracy.

Measurement of the perfusion-diffusion mismatch and calculation of infarction probability using MRI-based approaches for tissue-at-risk evaluation can be applied in stroke treatment decisions. Bouts et al. (47) analyzed the ability of five algorithms to depict potentially salvageable tissue using MRI imaging from rats subjected to a right-sided MCA occlusion without subsequent reperfusion, and with spontaneous or thrombolysis-induced reperfusion. The highest accuracy of risk-based identification of acutely salvageable ischemic tissue that could recover on subsequent reperfusion was observed using a generalized linear model (Dice's similarity index = 0.79 ± 0.14). Similarly, Huang et al. (48) used an SVM to predict infarct on a pixel-by-pixel basis using acute cerebral blood flow (CBF) and apparent diffusion coefficient (ADC) on MRI data. Serial images were collected during the acute phase up to 3 h and again at 24 h from 12 rats in each of the stroke groups exposed to a 30-min, 60-min, or permanent middle cerebral artery (MCA) occlusion. The accuracy observed for this approach was high in all groups and was enhanced by adding neighboring pixel information and spatial infarction incidence.

Takahashi et al. (44) designed a method to identify a hyperdense MCA, also known as the MCA dot sign, an important evaluation in an NCHCT as it represents a thrombus in a vessel. The authors created ROIs around the Sylvian fissure region and identified MCA dots based on the morphologic top-hat transformation, and classified images using an SVM with four features. Two hundred and ninety-seven CT images from seven patients with an MCA dot sign were classified by an SVM system, which exhibited a maximum sensitivity of 97.5% at a false positive rate of 1.28 per image and 0.5 per hemisphere while assessing the MCA dot sign.

Another application of ML in AIS is predicting factors that will contribute to neurological deterioration and increased morbidity, such as cerebral edema. Chen et al. (49) proposed a machine learning algorithm using serial CT scans of stroke patients to delineate and measure cerebrospinal fluid (CSF) volume over time, as it may represent a sensitive biomarker of cerebral edema progression. The initial cohort consisted of 155 subjects and preliminary processing using a generalized estimating equations (GEE) model top to calculate CSF volumes over time, adjusting for age, demonstrated that a reduction in CSF volume from baseline to final CT was correlated with infarct volume, the presence of cerebral edema, and the degree of midline shift. Comparatively, Dhar et al. (50) validated an automated technique for intracranial CSF segmentation by an ensemble of RF-based machine learning with a geodesic active contour (GAC) segmentation. CSF spaces were outlined on scans performed within 6 h of stroke onset and then closest to 24 h later in 38 patients. This method accurately tracked changes in CSF volume with an average DSC > 0.7. Pearson correlation coefficients between the changes in CSF and the ground truth were found to be statistically significant. These algorithms represent a potential for future research and may serve as a biomarker of cerebral edema severity.

The outcome of acute ischemic stroke patients is dependent on therapy, and risks for complications should be considered when deciding for stroke therapy. Yu et al. (53) established a method to predict the location and extent of hemorrhagic transformation (HT) in stroke, the most severe complication following reperfusion therapy. PWI and DWI of 165 patients treated with reperfusion therapy in a stroke center were collected and analyzed using five machine learning approaches, with Kernel spectral regression exhibiting an accuracy of 83.7 ± 2.6%. A multi-center retrospective study (52) assessed the predictive power for hemorrhagic transformation of PWI in MRI. Dynamic T2- weighted perfusion MR images from 263 patients from four medical centers were collected and served as input for linear and nonlinear predictive models, the latter having an average accuracy >85% in predicting HT. In one study, Nielsen et al. (54) ran a deep learning convoluted neural network (CNN_deep_) with 9 biomarkers as input to calculate lesion volume in patients treated with IV tPA. Input data from 29 untreated patients and 35 patients that received IV tPA were compared. This model predicted final infarct volume with 88% accuracy, being superior to other models in this study. Bentley et al. (51) predicted the risk of symptomatic intracerebral hemorrhage (sICH) after IV thrombolysis therapy. CT images of 116 patients who were treated with IV tPA, 16 of which had sICH, were entered as inputs into an SVM along with clinical severity. They found a better prognostication of the SVM when compared to the traditional clinician-based prognostication tools such as Hemorrhage after thrombolysis (HAT), and Sugar, Early Infarct signs, Dense cerebral artery sign, Age, and NIHSS scores (SEDAN).

Machine learning algorithms based on structural and functional MR images as input may assist in predicting motor deficits in stroke patients. Forkert et al. (56) applied 12 SVM classification models in calculating the corresponding 30-day mRS score of ischemic stroke patients through parameters including lesion overlap from different brain regions, stroke laterality, and other optional features such as infarct volume, NIHSS at admission, and patient age. Superior mRS prediction was observed by integrating the optional features and providing stroke location information, with a multi-value mRS prediction accuracy of 56%, and a dichotomized mRS (0–2 vs. 3–5) prediction accuracy of 85%. In a study by Rondina et al. (57), a proposed model to predict upper extremity motor deficit in 50 stroke patients was developed from data on structural MRI instead of functional MRI. Lesion probability images were derived using patterns of voxels and was then compared to lesion load per ROI in predicting outcomes, with the former providing better results when multiple regions of interest such as a range of cortical and subcortical motor areas and corticospinal tract were analyzed.

## Current challenges and future directions in machine learning for acute ischemic stroke

Early promising results have demonstrated that ML techniques may be useful as decision support tools in treatment choices for AIS. To improve the generalizability of the findings discussed above, however, there are a number of limitations in currently existing architectures that need to be addressed. The first limitation is that of sample size. Deep learning algorithms using medical imaging often require datasets of tremendous magnitude, the types of which may not be readily available. For example, an ML algorithm demonstrated superior performance at differentiating skin cancer lesions from their benign corresponding equivalent when compared against 21 board-certified dermatologists, using a dataset of nearly 130,000 images (58). A dataset of this size in AIS for public use does not currently exist. This shortcoming, however, has been recognized as a problem that can and ought to be solved, and multiple calls for the creation of such a repository have been made (59). The obstacles in inter-institutional data sharing, as well as a lack of funding to correctly pre-process and curate these images, along limitations to host such a dataset account for some of the delays in the creation of this repository.

Another limitation encountered in neuroimaging-based ML techniques is the need for labeling regions of interest or “gold standard” findings on the images. That is to say, beyond collecting the images, the images and the findings on the images would need to be identified for the question being evaluated. For example, a study evaluating the presence or absence of a hyperdense MCA would need each image to be tagged with the true result, to train the algorithm. Without foresight, this degree of manual curating could be required for each individual project.

## Conclusion

Machine learning applications are expanding in the medical field for diagnostic and therapeutic purposes, and the rapidly expanding and increasingly neuro-imaging reliant field of AIS is proving to be fertile ground. There is a particular need for ML solutions in this field, which is faced with the challenge of increasingly complex data, with limited human expert resources. Future directions in ML for AIS may require collaborative approaches across multiple institutions to build a robust dataset for efficient training of ML networks.

## Author contributions

HK and SS contributed equally to manuscript conception, design, revisions, and approved the submitted version. VL contributed to the manuscript design, revisions and edits and approved the submitted version.

### Conflict of interest statement

The authors declare that the research was conducted in the absence of any commercial or financial relationships that could be construed as a potential conflict of interest.
